# Persistent disparities in diabetic retinopathy outcomes among socially deprived individuals despite treatment adherence

**DOI:** 10.1038/s41433-025-04071-y

**Published:** 2025-10-08

**Authors:** Alexander T. Hong, Jason S. Chwa, Lucas Humayun, Hossein Ameri

**Affiliations:** 1 Keck School of Medicine, University of Southern California, Los Angeles, CA USA; 2 Department of Ophthalmology, USC Roski Eye Institute, Keck School of Medicine, University of Southern California, Los Angeles, CA USA

**Keywords:** Outcomes research, Retinal diseases

## Abstract

**Objective:**

To evaluate the long-term impact of social determinants of health on diabetic retinopathy (DR) incidence, complications, and management in patients with type 2 diabetes mellitus (T2DM), with emphasis on disparities among treatment-adherent individuals and demographic subgroups.

**Methods:**

We conducted a retrospective cohort study using TriNetX, a national electronic health records network spanning 70 U.S. healthcare organisations. Adults diagnosed with T2DM were categorised into socially deprived and non-deprived cohorts based on ICD-10 codes related to housing instability, food insecurity, and financial hardship. Propensity score matching balanced cohorts on demographics, comorbidities, laboratory values, medications, and ophthalmic care utilisation. Outcomes included incident DR, sight-threatening complications, ophthalmic treatment and diagnostics, and documented treatment nonadherence. Cox proportional hazards models estimated hazard ratios (HRs) with 95% confidence intervals (CIs).

**Results:**

Matched cohorts included 62,786 socially deprived and 62,786 non-deprived patients (mean age, 54 years; 42% female; 49% White, 27% Black, 14% Hispanic). Over a 10-year follow-up, social deprivation was associated with increased risk of DR (HR 1.40, 95% CI 1.34–1.47) and sight-threatening complications, including blindness. Documented nonadherence was significantly increased in the socially deprived cohort (HR 3.57, 95% CI 3.47–3.68). Among patients without documented nonadherence, social deprivation was associated with increased DR risk (HR 1.44, 95% CI 1.35–1.54) and treatment utilisation. Disparities were most pronounced in males, Hispanic individuals, and adults aged 18–39.

**Conclusions:**

Social deprivation, regardless of documented treatment adherence, increased DR incidence and complications. Targeted interventions are needed to address persistent disparities and reduce DR burden.

## Introduction

Diabetic retinopathy (DR), a prevalent and serious sequela of diabetes mellitus, affects approximately 26% of individuals with diabetes in the United States [1]. Despite advancements in therapy, such as intravitreal anti-VEGF injections (IVI) and panretinal photocoagulation (PRP), DR remains a leading cause of vision impairment [2, 3]. While poor glycaemic control, longer diabetes duration, and coexisting microvascular complications are well established risk factors, emerging evidence highlights the significant influence of social determinants of health (SDOH) on DR incidence, progression, and treatment utilisation [4, 5].

Socially deprived individuals—including those with food insecurity, housing instability, and low income—are more likely to present with advanced-stage DR and have an increased risk of sight-threatening complications [6, 7]. However, the reasons for more advanced disease progression remain poorly understood. While prior studies have shown socially disadvantaged individuals experience lower rates of DR screening and ophthalmic care utilisation, it remains unclear whether these disparities are driven solely by barriers to care or whether underlying structural inequities contribute to worse outcomes even amongst individuals who access and remain compliant with treatment [8–10].

Existing research often attributes disparities in DR outcomes to screening and treatment nonadherence, but this assumption has not been rigorously tested [11–13]. Additionally, subgroup-specific risks remain underexplored, particularly whether racial and ethnic minority groups, age, and sex intersect with social deprivation and treatment adherence in DR outcomes. To address these gaps, we leveraged a comprehensive national database to investigate the independent and combined effects of social deprivation, race, age, and sex on DR progression, access to ophthalmic care, and sight-threatening complications, with a focus on assessing whether disparities persist independent of treatment nonadherence.

## Methods

### Study design

This retrospective cohort study used the U.S. Collaborative Network within the TriNetX Analytics platform (TriNetX, Cambridge, Massachusetts, USA). TriNetX is a federated health research network providing access to aggregated de-identified electronic health record (EHR) data from over 118 million patients across 70 U.S. healthcare organisations [14]. The study was deemed exempt by the University of Southern California Institutional Review Board (HS-24-00615), with informed consent waived due to the use of de-identified data. The study adhered to the Strengthening the Reporting of Observational Studies in Epidemiology (STROBE) guidelines and the tenets of the Declaration of Helsinki [15].

**Fig. 1:**
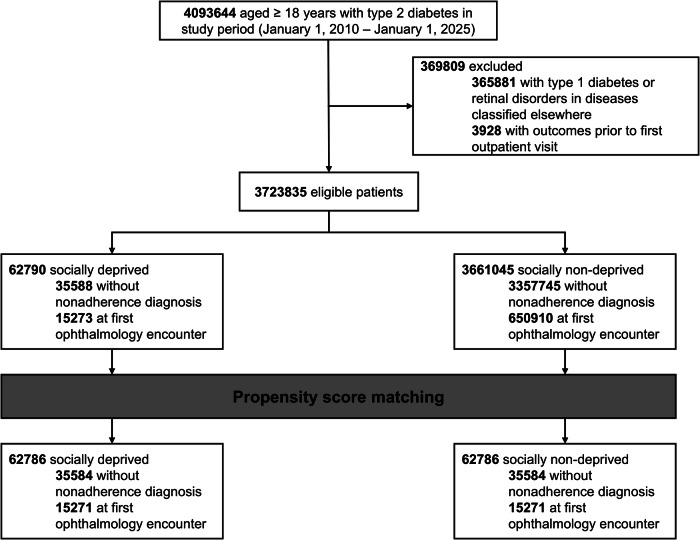
Cohort selection flow diagram. Study flowchart of selection criteria for study cohort of patients with type 2 diabetes, stratified by socially deprived and nondeprived cohorts.

Patients with type 2 diabetes mellitus (T2DM) were identified using the *International Classification of Diseases, 10th Revision* (ICD-10) code E11. Eligible participants included adults (≥18 years) with a confirmed diagnosis of T2DM between January 1, 2010, and January 1, 2025 with a registered outpatient visit as analysis was run in January 2025 (Fig. 1). Cohorts were split into two groups: (1) those who were socially deprived, identified using ICD-10 codes for homelessness (Z59.0), inadequate housing (Z59.1), problems related to health literacy (Z55.6), lack of adequate food (Z59.4), extreme poverty (Z59.5), or low income (Z59.6); and (2) those classified as non-deprived, with no records containing these codes [16, 17]. Patients were further stratified to a documented adherence cohort, identified as lack of documented diagnoses for nonadherence to medications (Z91.12-14, Z91.19) and treatments (Z53.20, Z53.21) at any point following the index event. These codes were not specific to T2DM or DR-related treatment but have been validated in cohorts of patients with diabetes and are understood to reflect broader patterns of healthcare nonadherence [18]. Adherence status was fixed for the duration of follow-up and did not change dynamically over time (e.g., a patient coded as nonadherent at one year post-index was considered nonadherent for the entire duration of the analysis). Nonadherence was conceptualised as a stratification variable to determine whether disparities in outcomes associated with social deprivation persisted even amongst individuals with no documented adherence concerns. Individuals with a history of type 1 diabetes mellitus (ICD-10: E10), retinal disorders classified elsewhere (ICD-10: H36), or prior DR and sight-threatening complications were excluded. The first outpatient visit after diagnosis of T2DM was considered the index event.

### Outcome measures

The primary outcome was incident DR diagnoses, grouped into any DR, non-proliferative diabetic retinopathy (NPDR), or proliferative diabetic retinopathy (PDR). Secondary outcomes included sight-threatening complications, including retinal detachment (RD), vitreous haemorrhage (VH), macular oedema, and blindness or low vision. Ophthalmic utilisation was divided into treatments, including IVI, PRP or pars plana vitrectomy (PPV), or diagnostic imaging including dilated fundus examinations, optical coherence tomography (OCT) imaging, and fluorescein angiography. Additionally, the study assessed documented nonadherence to medications, procedures, or both. Patients with DR or sight-threatening outcomes prior to the index event were excluded. Patients were followed from the index date and right-censored at the earliest of the following: (1) occurrence of the outcome event (e.g., DR), (2) mortality, (3) loss to follow-up (defined as the end of available patient data in the TriNetX database), or (4) the end of the study period, up to 10 years post-index.

### Statistical analysis

All statistical analyses were performed using the built-in functions on TriNetX Analytics platform. Propensity score matching (PSM) was employed to reduce confounding and ensure comparability between groups. Covariates included age at index visit, sex (as identified in the EHR), race and ethnicity (as identified in the EHR), comorbidities (chronic kidney disease, hyperlipidaemia, hypertension, ischaemic heart disease), medication use (insulin, oral hypoglycaemic agents, lipid-modifying agents), and laboratory values (haemoglobin A1c, serum glucose, cholesterol, triglycerides, and body mass index). Billing codes for ophthalmology encounters (Current Procedural Terminology [CPT] codes 92002, 92004, 92012, and 92014) were also included to account for differences in care-seeking behaviour that might affect DR outcomes. Further details on the codes used in analysis are provided in Supplementary Table 1.

Continuous variables were expressed as mean (± standard deviation [SD]), while categorical variables were expressed as counts and percentages. PSM (1:1) employed the nearest neighbour greedy matching algorithm with a calliper of 0.1 standardised differences to create cohorts with matched characteristics. Covariate balance was evaluated using standardised mean differences (SMD), with values < 0.1 indicating well-balanced groups. Kaplan-Meier analysis was conducted to estimate survival probabilities after propensity matching, with log-rank tests used to estimate differences in survival between cohorts. Hazard ratios (HRs) with 95% confidence intervals (CIs) were calculated using Cox proportional hazards regression for all outcomes on propensity score matched cohorts. For each outcome, separate models were run with right-censoring at 1-, 5-, and 10-years post-index to estimate time-specific HRs, allowing for evaluation of risk across distinct follow-up intervals. Each outcome was analysed independently, with patients being eligible for inclusion in multiple models. The proportional hazards assumption was assessed using scaled Schoenfeld’s residuals within R’s Survival package (version 3.2-3). Statistical significance was set at P < 0.05 using two-sided tests.

Subgroup analyses explored potential interactions between demographics on DR outcomes, stratifying by sex (male vs. female), race/ethnicity (non-Hispanic White, non-Hispanic Black, non-White Hispanic, non-Hispanic Asian), and age group (18–39, 40–64, ≥65). To explore domain-specific effects, we conducted additional analyses within the socially deprived cohort by comparing three predefined subgroups: those experiencing financial hardship (poverty, low income, or food insecurity), housing instability, and health literacy barriers. Additionally, a sensitivity analysis evaluated adherent cohorts at the first ophthalmology encounter after T2DM diagnosis to evaluate whether disparities in outcomes persisted between deprived and non-deprived individuals within a subset of patients who had established ophthalmic care and documented adherence, thereby controlling for initial care participation and treatment engagement.

## Results

### Baseline characteristics

The initial cohorts consisted of 62,790 socially deprived patients and 3,661,045 non-deprived patients with T2DM. Before PSM, the socially deprived cohort was younger (mean ± [SD] age, 54 ± 13 vs. 60 ± 13 years) and had a lower proportion of females (42% vs 48%; P < 0.001). Racial and ethnic composition differed significantly with a lower proportion of White individuals (49% vs 61%; P < 0.001) and a higher proportion of Black or African American individuals (27% vs 15%; P < 0.001) and Hispanic or Latino individuals (14% vs 12%; P < 0.001). After PSM, both cohorts contained 62,786 individuals (mean [SD] age, 54 [13] years, 42% female, 49% White, 27% Black or African American, 14% Hispanic or Latino) and were well-balanced (Table 1). The mean follow-up duration was 4.6 ± 3.1 years in the socially deprived cohort and 5.0 ± 3.1 years in the non-deprived cohort. Follow-up at 1, 5, and 10 years was observed in 97.1%, 83.5%, and 69.2% of socially deprived patients, and 94.1%, 82.8%, and 70.4% of non-deprived patients, respectively.

**Table 1:** Baseline characteristics of patients with type 2 diabetes mellitus stratified by social deprivation status before and after propensity score matching.

Characteristic	Before propensity score matching, No. (%)			After propensity score matching, No. (%)		
	Socially deprived (N = 62,790)	Non-deprived (N = 3,661,045)	SMD	Socially deprived (N = 62,786)	Non-deprived (N = 62,786)	SMD
**Demographics**						
Age at index, years, mean ± SD	54.3 ± 13.1	60.0 ± 13.5	0.428	54.3 ± 13.1	54.3 ± 13.6	0.004
Female	26,525 (42.2)	1,773,734 (48.4)	0.125	26,525 (42.2)	26,364 (42.0)	0.005
White	30,955 (49.3)	2,236,650 (61.1)	0.239	30,955 (49.3)	30,652 (48.8)	0.01
American Indian or Alaska Native	423 (0.7)	10852 (0.3)	0.079	422 (0.7)	396 (0.6)	0.005
Native Hawaiian or Other Pacific Islander	1217 (1.9)	23827 (0.7)	0.114	1215 (1.9)	1179 (1.9)	0.004
Hispanic or Latino	8980 (14.3)	426,397 (11.6)	0.079	8977 (14.3)	8796 (14.0)	0.008
Black or African American	16,955 (27.0)	536,491 (14.7)	0.308	16,954 (27.0)	16,975 (27.0)	0.001
Asian	1735 (2.8)	155,056 (4.2)	0.08	1735 (2.8)	1561 (2.5)	0.017
**Comorbidities**						
Chronic kidney disease	8038 (12.8)	302,185 (8.3)	0.149	8034 (12.8)	7692 (12.3)	0.016
Hyperlipidaemia	21,489 (34.2)	953,065 (26.0)	0.179	21,487 (34.2)	21631 (34.5)	0.005
Ischaemic heart diseases	13,164 (21.0)	541,639 (14.8)	0.162	13,160 (21.0)	12,963 (20.6)	0.008
Hypertensive diseases	37,734 (60.1)	1,648,621 (45.0)	0.305	37,730 (60.1)	37,999 (60.5)	0.009
Chronic lower respiratory diseases	18,770 (29.9)	500,098 (13.7)	0.401	18,766 (29.9)	18,924 (30.1)	0.005
Tobacco use	7660 (12.2)	94,624 (2.6)	0.374	7656 (12.2)	7478 (11.9)	0.009
Cerebrovascular diseases	7473 (11.9)	269,969 (7.4)	0.154	7472 (11.9)	7130 (11.4)	0.017
Type 2 diabetes mellitus with diabetic neuropathy	2171 (3.5)	58121 (1.6)	0.119	2171 (3.5)	1969 (3.1)	0.018
Diseases of arteries, arterioles and capillaries	8196 (13.1)	293819 (8.0)	0.164	8192 (13.0)	7870 (12.5)	0.015
**Ophthalmology care utilisation**						
General ophthalmological services	5669 (9.0)	184100 (5.0)	0.157	5669 (9.0)	5625 (9.0)	0.002
**Medications**						
Lipid modifying agents	22,210 (35.4)	1,248,406 (34.1)	0.027	22,209 (35.4)	21,808 (34.7)	0.013
Insulins and analogues	17,188 (27.4)	684,195 (18.7)	0.207	17,185 (27.4)	16,956 (27.0)	0.008
Blood glucose lowering agents	16,524 (26.3)	1,043,894 (28.5)	0.049	16,523 (26.3)	15,964 (25.4)	0.02
**Laboratory values, mean ± SD**						
Haemoglobin A1c (%)	7.1 ± 2.2	7.1 ± 2.0	0.008	7.1 ± 2.2	7.1 ± 2.2	<0.001
BMI (kg/m^2^)	33.7 ± 9.4	33.3 ± 8.1	0.047	33.7 ± 9.4	34.2 ± 8.8	0.052
Total cholesterol (mg/dL)	173.7 ± 51.7	174.0 ± 50.8	0.008	173.7 ± 51.7	174.1 ± 51.4	0.009

*SMD* standardised mean difference, *SD* standard deviation, *BMI* body mass index.

At 1-year follow-up, DR incidence was higher in the socially deprived cohort (2.8% vs. 2.1%), with increased disparity at 10 years (7.5% vs. 4.9%, P < 0.001) (Supplementary Fig. 1, Supplementary Table 2). Social deprivation was associated with increased DR risk at 1- (HR 1.25, 95% CI 1.17–1.34), 5- (HR 1.30, 95% CI 1.24–1.37), and 10 years (HR 1.40, 95% CI 1.34–1.47) compared to those without deprivation (Table 2). Risks for NPDR and PDR remained consistently elevated across timepoints in the socially deprived cohort (NPDR at 10 years: HR 1.59, 95% CI 1.49–1.70; PDR at 10 years: HR 1.61, 95% CI 1.44–1.80). Socially deprived patients showed increased risks for vision-threatening complications including RD, macular oedema, VH, and blindness or low vision across all time periods.

**Table 2:** Effect of social deprivation on diabetic retinopathy outcomes in patients with type 2 diabetes mellitus over 1-, 5-, and 10-year follow-up.

Outcome	1 year	5 years	10 years
	HR (95% CI)	HR (95% CI)	HR (95% CI)
*DR incidence*			
Any DR	1.25 (1.17, 1.34)	1.30 (1.24, 1.37)	1.40 (1.34, 1.47)
NPDR	1.52 (1.33, 1.73)	1.51 (1.39, 1.63)	1.59 (1.49, 1.70)
PDR	1.33 (1.09, 1.62)	1.63 (1.43, 1.85)	1.61 (1.44, 1.80)
*Sight-threatening complications*			
RD	1.35 (1.06, 1.71)	1.35 (1.17, 1.57)	1.40 (1.23, 1.60)
VH	1.24 (0.96, 1.60)	1.75 (1.48, 2.05)	1.63 (1.42, 1.88)
Blindness or low vision	1.96 (1.72, 2.24)	1.98 (1.83, 2.15)	2.07 (1.92, 2.22)
Macular oedema	1.18 (1.01, 1.37)	1.39 (1.26, 1.53)	1.42 (1.30, 1.54)
*DR treatment*			
IVI	1.28 (1.08, 1.52)	1.33 (1.17, 1.52)	1.49 (1.33, 1.67)
PRP	1.02 (0.82, 1.26)	1.37 (1.16, 1.61)	1.48 (1.28, 1.70)
PPV	1.15 (0.91, 1.46)	1.39 (1.18, 1.65)	1.53 (1.32, 1.78)
*Diagnostic imaging*			
OCT	1.24 (1.13, 1.35)	1.32 (1.24, 1.41)	1.41 (1.33, 1.48)
Fundus photography	1.14 (1.00, 1.30)	1.22 (1.12, 1.32)	1.22 (1.14, 1.31)
Fluorescein angiography	1.10 (0.84, 1.42)	1.27 (1.07, 1.51)	1.34 (1.15, 1.55)
*Nonadherence to treatment*			
Nonadherence (Overall)	3.94 (3.75, 4.13)	3.26 (3.17, 3.36)	3.57 (3.47, 3.68)
Nonadherence to medications	3.93 (3.68, 4.19)	3.46 (3.32, 3.60)	3.89 (3.73, 4.05)
Nonadherence to procedures	3.59 (3.27, 3.94)	2.85 (2.70, 3.01)	3.15 (3.00, 3.32)

*CI* confidence interval, *DR* diabetic retinopathy, *HR* hazard ratio, *IVI* intravitreal injection, *NPDR* non-proliferative diabetic retinopathy, *OCT* optical coherence tomography, *PDR* proliferative diabetic retinopathy, *PPV* pars plana vitrectomy, *PRP* panretinal photocoagulation, *RD* retinal detachment, *VH* vitreous haemorrhage.

Ophthalmic treatment and diagnostic utilisation was comparable in the short-term between cohorts (Table 2). However, at 5- and 10-year follow-up, socially deprived patients had significantly greater utilisation of IVI, PRP, PPV, and diagnostic imaging. Additionally, documented nonadherence to medications and procedures was markedly higher in the socially deprived cohort at 1 year (HR 3.94, 95% CI 3.75–4.13) and persisted at 5- (HR 3.26, 95% CI 3.17–3.36) and 10 years (HR 3.57, 95% CI 3.47–3.68).

### Adherence cohort analysis

To assess whether disparities extended beyond documented treatment nonadherence, we conducted a stratified analysis excluding nonadherent patients. After matching, 35,584 patients remained in each cohort (mean age 56 ± 13 years; 44% female; 50% White, 23% Black, 15% Hispanic) with balanced baseline characteristics (Table 3). At 1-year follow-up, DR incidence was higher in the socially deprived cohort (2.6% vs. 2.0%), with increased disparity at 10 years (6.8% vs. 4.3%, P < 0.001) (Supplementary Fig. 2, Supplementary Table 3). Social deprivation remained significantly associated with elevated risks of DR, NPDR, PDR, and sight-threatening complications at 1, 5, and 10 years, independent of documented treatment adherence (Table 4). While ophthalmic intervention and diagnostic use were similar at 1 year, ophthalmic care utilisation increased significantly amongst the socially deprived by 5 and 10 years.

**Table 3:** Baseline characteristics of patients with type 2 diabetes mellitus without documented nonadherence stratified by social deprivation status before and after propensity score matching.

	Before propensity score matching, No. (%)			After propensity score matching, No. (%)		
Characteristic	Socially deprived (N = 35,588)	Non-socially deprived (N = 3,357,745)	SMD	Socially deprived (N = 35,584)	Non-socially deprived (N = 35,584)	SMD
**Demographics**						
Age, years, mean ± SD	55.7 ± 13.4	60.3 ± 13.4	0.347	55.7 ± 13.4	55.6 ± 13.7	0.007
Female	15,666 (44.0)	1,625,978 (48.4)	0.088	15,665 (44.0)	15,658 (44.0)	<0.001
White	17,875 (50.2)	2,063,147 (61.4)	0.227	17,875 (50.2)	17,651 (49.6)	0.013
American Indian or Alaska Native	226 (0.6)	9797 (0.3)	0.051	225 (0.6)	207 (0.6)	0.007
Native Hawaiian or Other Pacific Islander	372 (1.0)	19,072 (0.6)	0.053	372 (1.0)	393 (1.1)	0.006
Hispanic or Latino	5639 (15.8)	388,780 (11.6)	0.124	5637 (15.8)	5431 (15.3)	0.016
Black or African American	8306 (23.3)	466,813 (13.9)	0.244	8303 (23.3)	8298 (23.3)	<0.001
Asian	958 (2.7)	144,830 (4.3)	0.088	958 (2.7)	953 (2.7)	0.001
**Comorbidities**						
Chronic kidney disease	3581 (10.1)	258,539 (7.7)	0.083	3580 (10.1)	3455 (9.7)	0.012
Hyperlipidaemia	11,526 (32.4)	845,863 (25.2)	0.159	11,526 (32.4)	11,503 (32.3)	0.001
Ischaemic heart diseases	5936 (16.7)	470,701 (14.0)	0.074	5936 (16.7)	5649 (15.9)	0.022
Hypertensive diseases	19,768 (55.5)	1,469,595 (43.8)	0.237	19,767 (55.6)	19,817 (55.7)	0.003
Chronic lower respiratory diseases	9059 (25.5)	425,862 (12.7)	0.330	9055 (25.4)	9065 (25.5)	0.001
Tobacco use	3046 (8.6)	72,982 (2.2)	0.286	3042 (8.5)	2943 (8.3)	0.010
Cerebrovascular diseases	3528 (9.9)	232,996 (6.9)	0.107	3528 (9.9)	3423 (9.6)	0.010
Type 2 diabetes mellitus with diabetic neuropathy	873 (2.5)	48,506 (1.4)	0.073	873 (2.5)	773 (2.2)	0.019
Diseases of arteries, arterioles and capillaries	3983 (11.2)	254,134 (7.6)	0.125	3983 (11.2)	3813 (10.7)	0.015
**Ophthalmology care utilisation**						
General ophthalmological services	3363 (9.4)	164,822 (4.9)	0.177	3360 (9.4)	3365 (9.5)	<0.001
**Medications**						
Lipid modifying agents	11,649 (32.7)	1,132,758 (33.7)	0.021	11,649 (32.7)	11,391 (32.0)	0.015
Insulins and analogues	7748 (21.8)	596,927 (17.8)	0.100	7748 (21.8)	7538 (21.2)	0.014
Blood glucose lowering agents	8753 (24.6)	958,088 (28.5)	0.089	8753 (24.6)	8479 (23.8)	0.018
**Laboratory values, mean ± SD**						
Hemoglobin A1c (%)	7.1 ± 2.1	7.1 ± 1.9	0.026	7.1 ± 2.1	7.1 ± 2.1	0.008
BMI (kg/m^2^)	34.2 ± 9.2	33.2 ± 8.0	0.108	34.2 ± 9.2	34.0 ± 8.5	0.013
Total cholesterol (mg/dL)	176.7 ± 49.9	174.0 ± 50.4	0.054	176.7 ± 49.9	176.0 ± 51.1	0.015

*SMD* standardised mean difference, *SD* standard deviation, *BMI* body mass index.

**Table 4:** Effect of social deprivation on diabetic retinopathy outcomes in patients with type 2 diabetes mellitus without documented nonadherence over 1-, 5-, and 10-year follow-up.

Outcome	1 year	5 years	10 years
	HR (95% CI)	HR (95% CI)	HR (95% CI)
*Diabetic retinopathy incidence*			
Any DR	1.25 (1.13, 1.38)	1.35 (1.26, 1.44)	1.44 (1.35, 1.54)
NPDR	1.59 (1.32, 1.91)	1.57 (1.41, 1.75)	1.75 (1.59, 1.92)
PDR	1.45 (1.10, 1.92)	1.68 (1.39, 2.02)	1.72 (1.46, 2.03)
*Sight-threatening complications*			
RD	1.52 (1.09, 2.11)	1.45 (1.17, 1.78)	1.32 (1.10, 1.58)
VH	1.51 (1.06, 2.16)	1.86 (1.49, 2.34)	1.81 (1.47, 2.23)
Blindness or low vision	1.73 (1.44, 2.07)	1.79 (1.60, 2.00)	1.97 (1.78, 2.18)
Macular oedema	1.13 (0.92, 1.40)	1.46 (1.28, 1.67)	1.43 (1.27, 1.61)
*DR treatment*			
IVI	1.02 (0.83, 1.25)	1.42 (1.19, 1.68)	1.33 (1.15, 1.55)
PRP	1.22 (0.89, 1.67)	1.49 (1.16, 1.92)	1.58 (1.27, 1.97)
PPV	0.93 (0.69, 1.26)	1.21 (0.96, 1.52)	1.26 (1.03, 1.53)
*Diagnostic imaging*			
OCT	1.23 (1.09, 1.38)	1.44 (1.32, 1.56)	1.44 (1.34, 1.54)
Fundus	1.09 (0.92, 1.29)	1.34 (1.20, 1.51)	1.23 (1.12, 1.35)
Fluorescein angiography	0.96 (0.68, 1.36)	1.34 (1.06, 1.69)	1.33 (1.08, 1.63)

*CI* confidence interval, *DR* diabetic retinopathy, *HR* hazard ratio, *IVI* intravitreal injection, *NPDR* non-proliferative diabetic retinopathy, *OCT* optical coherence tomography, *PDR* proliferative diabetic retinopathy, *PPV* pars plana vitrectomy, *PRP* panretinal photocoagulation, *RD* retinal detachment, *VH* vitreous haemorrhage.

### Subgroup and sensitivity analysis

At 10-year follow-up, stratified analyses by sex, age, and race/ethnicity revealed persistent disparities amongst adherent socially deprived individuals. Social deprivation was linked to higher DR incidence, sight-threatening complications, and intervention use in both men and women (Supplementary Table 4). Racial disparities showed the highest DR risk amongst Hispanic individuals, and the lowest amongst non-Hispanic Black individuals (Supplementary Table 5). Risk of developing PDR was highest amongst non-Hispanic Asian individuals and lowest amongst non-Hispanic White individuals. All age groups showed elevated DR risk, most notably in the 18–39 age group, though this youngest cohort had lower rates of complications and similar intervention use across deprivation status (Supplementary Table 6).

Amongst the three domains of social deprivation, financial hardship was most consistently associated with higher risk of incident diabetic retinopathy and sight-threatening complications when compared to both housing instability and low health literacy (Supplementary Table 7). Documented nonadherence was greatest amongst individuals experiencing housing instability. Low health literacy appeared to increase the risk of DR development but did not significantly affect the risk of sight-threatening complications compared to housing instability.

Sensitivity analysis followed documented adherent individuals at their first ophthalmology visit post-T2DM diagnosis (Supplementary Table 8). At 1 and 5 years, DR risk did not differ by social deprivation; however, by 10 years, DR incidence and treatment use were significantly higher in the deprived cohort (Supplementary Table 9).

## Discussion

This study demonstrates that social deprivation is independently associated with higher incidence and greater severity of diabetic retinopathy, even amongst patients with documented adherence to medications and treatments. Notably, despite comparable or greater ophthalmic intervention utilisation, socially deprived individuals continued to experience disproportionate risks of DR progression and sight-threatening complications. These disparities persisted across all demographic subgroups, underscoring the influence of systemic factors beyond care access or individual adherence on long-term DR outcomes.

While prior studies have highlighted barriers to DR screening and follow-up in socially deprived populations, our findings add to the existing literature by demonstrating that disparities persist even when these individuals access care and adhere to treatment [8, 9, 12, 19]. Socially deprived patients had greater utilisation of IVI, PRP, and PPV and diagnostic screening, but still experienced higher risks of progression to sight-threatening complications, likely to reflect more advanced disease at presentation. This suggests that higher intervention rates in these groups stem from delayed diagnoses and more severe baseline pathology, not enhanced disease control. Indeed, social deprivation more than doubles the risk of missed screenings, contributing to late-stage presentation [20, 21]. Even post-diagnosis, barriers such as delayed treatment escalation, inconsistent care intensity, and fragmented healthcare delivery may further compound disease progression [12, 20, 22]. These disparities are especially troubling given that early DR treatment can prevent up to 90% of severe vision loss [23, 24]. Yet, studies have shown that individuals with low SDOH remain significantly less likely to receive timely, guideline-concordant care [25, 26]. Addressing these systemic gaps requires targeted interventions that address not only medical management but also the structural and social barriers that perpetuate inequities in DR outcomes.

Stratification by sex, race/ethnicity, and age revealed that social deprivation is a persistent determinant of DR incidence and progression amongst adherent patients with T2DM. While prior studies often attribute disparities to lower healthcare utilisation amongst racial and ethnic minorities, our findings indicate that social deprivation itself—independent of care access—drives worse outcomes across all racial and ethnic groups [27–29]. Similarly, while some studies propose that women may be at greater risk for DR-related complications due to lower healthcare engagement, we observed comparable risks amongst socially deprived men and women who accessed and adhered to treatment [30, 31]. Age-related assumptions that younger adults are less adherent were also challenged: young socially deprived adults had DR risks similar to older peers, underscoring that social disadvantage may outweigh age-related engagement patterns [32–34]. In other words, the adverse influence of social disadvantage can supersede age-related patterns in care engagement, highlighting the need to address social determinants alongside traditional risk factors. Additionally, we found that individuals experiencing financial hardship had a higher risk of incident diabetic retinopathy, while those with housing instability were more likely to have documented nonadherence. These findings align with prior studies showing that food and housing insecurity are associated with reduced engagement in eye care, suggesting potential downstream consequences on disease detection and management [9]. The elevated nonadherence observed amongst individuals with housing instability may reflect structural barriers such as limited transportation or inadequate access to pharmacies in medically underserved areas critical targets for future intervention. Given the intricate interplay of demographic and socioeconomic factors, future efforts to reduce DR disparities should prioritise intersectional, personalised care strategies.

The persistence of DR disparities despite healthcare engagement highlights serious public health and economic consequences, particularly for socially deprived populations. Delayed diagnosis and inadequate treatment escalation not only accelerate irreversible vision loss but also drive substantial healthcare expenditures, with common complications such as macular oedema requiring annual management costs exceeding $6000 per person and vision-related disability contributing to a staggering $120 billion economic burden [35, 36]. Our findings further emphasise the urgency of this issue, demonstrating that socially deprived individuals face significantly elevated risks of blindness and severe vision loss as early as one-year, with these disparities increasing over time. These early and progressive visual impairments may have profound consequences as nearly half of U.S. adults with severe NPDR or PDR report difficulty with visual tasks, exacerbating social isolation, financial strain, and diminished workforce participation [37, 38]. These inequities perpetuate cycles of economic instability, disproportionately burdening low SDOH communities through lost productivity and diminished quality of life [39, 40]. As vision impairment remains a leading cause of preventable disability amongst working-age adults, closing these gaps in DR care is not just a clinical priority but an urgent economic and public health imperative.

Addressing these disparities requires systemic reforms that extend beyond improving access and treatment attendance alone. Equitable treatment escalation should prioritise adherence to evidence-based protocols, with consistent monitoring and intervention intensity irrespective of socioeconomic status. Integrating SDOH screening into ophthalmology and primary care, leveraging EHRs to flag high-risk patients, and using automated referral systems can support earlier intervention [41–43]. Culturally responsive care models and a more diverse clinical workforce are equally essential, as patient-provider concordance in language, race, or culture may improve patient outcomes [44, 45]. Scalable technologies like teleophthalmology and AI-assisted DR screening can further streamline early detection and access to specialty care [46, 47]. Finally, interdisciplinary collaboration amongst ophthalmologists, primary care physicians, and endocrinologists, in addition to robust patient education, can help bridge care gaps, improve coordination, and ultimately reduce preventable vision loss.

Several limitations merit consideration. First, the retrospective design introduces potential for selection and information biases, and residual confounding may persist despite PSM. We attempted to address detection bias by including ophthalmologic CPT codes, but binary classification and overlapping billing practices may have limited our ability to fully capture the frequency and quality of ophthalmic care. EHR codes introduces potential coding errors, misclassification, and underreporting, including the codes used to define SDOH and documented nonadherence, which may be inconsistently recorded across institutions due to varying documentation practices, institutional workflows, or implicit provider biases [48, 49]. As our classification relied on the presence or absence of a documented nonadherence code, individuals not explicitly coded as nonadherent were assumed to be adherent. This approach inherently limits the ability to detect nonadherence that goes undocumented in clinical records. Adherence status was fixed over time and did not account for temporal changes in behaviour, such as intermittent lapses followed by re-engagement. As a result, both the prevalence and impact of social deprivation and nonadherence may be underestimated in this analysis. Furthermore, TriNetX is an aggregated database that limits access to patient-level data, restricting the ability to perform advanced handling of missing data or to directly assess loss to follow-up. While we accounted for missing laboratory values by including a separate category during propensity score matching, more nuanced imputation strategies were not feasible within the platform. Additionally, some diagnostic or treatment codes (e.g., VH, macular oedema, RD, IVI) may be attributed to ocular conditions other than diabetic retinopathy, potentially leading to outcome misclassification. While we attempted to mitigate this by excluding patients with pre-existing retinal diagnoses at baseline, the lack of indication may limit the precision of DR-related outcome attribution. Finally, as the database includes only patients who engage with healthcare systems, it may overrepresent those with higher disease burden and access, underestimating disparities amongst more marginalised populations.

## Conclusions

In this large, retrospective cohort study, we demonstrate that social deprivation is a significant determinant of DR incidence, progression, and sight-threatening complications in patients with T2DM, even amongst those with documented adherence to treatment. Despite greater use of ophthalmic interventions, socially deprived patients experienced significantly worse long-term DR outcomes, underscoring the limitations of healthcare engagement alone in mitigating disparities. These findings highlight the urgent need for targeted strategies that integrate SDOH into DR care, including enhanced screening, timely intervention, and cross-specialty coordination.

## Summary

### What was known before

- Social deprivation is associated with worse ocular outcomes in patients with diabetic retinopathy (DR)
- Prior studies have shown disparities in DR outcomes by socioeconomic status and ethnicity, often attributing these differences to poor adherence to eye care.

### What this study adds

- Social deprivation independently increases the risk of DR development and progression in patients with type 2 diabetes, even among those with treatment adherence.
- Patients from deprived backgrounds received equal or greater ophthalmic interventions yet continued to experience worse DR outcomes compared to less deprived peers.
- Disparities in DR progression persisted across age, sex, and race/ethnicity subgroups in deprived compared to non-deprived, emphasising the need for targeted public health interventions beyond clinical management.

## Supplementary information

Supplementary Online Content

## Data Availability

The data supporting the findings of this study were obtained under license from TriNetX, LLC and are subject to third-party restrictions. Access may be requested to the TriNetX research network by contacting TriNetX (https://live.trinetx.com). Access may require licensing fees, a formal data-sharing agreement, and adherence to privacy regulations, ensuring no identifiable patient information is disclosed.
