# Supplementary material for: Persistent disparities in diabetic retinopathy outcomes among socially deprived individuals despite treatment adherence
Source: Eye (Lond). 2025 Oct 8;39(18):3269–77. doi: 10.1038/s41433-025-04071-y (PMC12669577; doi:10.1038/s41433-025-04071-y)
Supplement: Supplementary file 1 — Supplementary Online Content [file 41433_2025_4071_MOESM1_ESM.docx]

**Supplementary Online Content**

Hong AT, Chwa JS, Humayun L, Ameri H. Persistent Disparities in Diabetic Retinopathy Incidence, Progression, and Management Among Socially Deprived Individuals Despite Treatment Adherence

**Supplementary Table 1.** Demographic, Diagnostic, Procedure, Medication, Laboratory, and Outcome Codes Used in Analysis

**Supplementary Table 2.** Social Deprivation and Cumulative Incidence Rates of Diabetic Retinopathy Outcomes in 62,786 Patients with Type 2 Diabetes

**Supplementary Table 3.** Social Deprivation and Cumulative Incidence Rates of Diabetic Retinopathy Outcomes in 35,584 Patients with Type 2 Diabetes and Documented Adherence

**Supplementary Table 4.** Effects of Social Deprivation and Sex on Diabetic Retinopathy Outcomes in Patients with Type 2 Diabetes over 10-Year Follow-Up

**Supplementary Table 5.** Effects of Social Deprivation and Race/Ethnicity on Diabetic Retinopathy Outcomes in Patients with Type 2 Diabetes over 10 Year Follow-Up.

**Supplementary Table 6**. Effects of Social Deprivation and Age on Diabetic Retinopathy Outcomes in Patients with Type 2 Diabetes over 10-Year Follow-Up

**Supplemental Table 7.** Effect of Social Deprivation on Diabetic Retinopathy Outcomes in Patients with Type 2 Diabetes Mellitus over 1-, 5-, and 10-year follow-up

**Supplementary Table 8.** Baseline Characteristics of Patients with Type 2 Diabetes Mellitus at First Ophthalmology Encounter Stratified by Social Deprivation Status Before and After Propensity Score Matching

**Supplementary Table 9.** Effect of Social Deprivation on Diabetic Retinopathy Outcomes in Patients with Type 2 Diabetes Mellitus Without Documented Nonadherence at First Ophthalmology Encounter over 1-, 5-, and 10-year follow-up

**Supplementary Figure 1.** Diabetic Retinopathy–Free Survival at 3 Years in Patients With Type 2 Diabetes

**Supplementary Figure 2.** Diabetic Retinopathy–Free Survival at 3 Years in Patients With Type 2 Diabetes and Documented Adherence

This supplementary material has been provided by the authors to give readers additional information about their work.

| **Supplementary Table 1. Demographic, Diagnostic, Procedure, Medication, Laboratory, and Outcome Codes Used in Analysis** | | |
| --- | --- | --- |
| **Category** | **Code** | **Description** |
| Demographics |  | |
|  | AI (TriNetX curated) | Age at Index |
|  | F (TriNetX curated) | Female |
|  | 2106-3 (TriNetX curated) | White |
|  | 1002-5 (TriNetX curated) | American Indian or Alaska Native |
|  | 2076-8 (TriNetX curated) | Native Hawaiian or Other Pacific Islander |
|  | 2135-2 (TriNetX curated) | Hispanic or Latino |
|  | 2054-5 (TriNetX curated) | Black or African-American |
|  | 2028-9 (TriNetX curated) | Asian |
| Diagnosis |  |  |
|  | N18 | Chronic kidney disease (CKD) |
|  | E78.5 | Hyperlipidaemia, unspecified |
|  | I20-I25 | Ischaemic heart diseases |
|  | I10-I1A | Hypertensive diseases |
|  | J40-J4A | Chronic lower respiratory diseases |
|  | Z72.0 | Tobacco use |
|  | I60-I69 | Cerebrovascular diseases |
|  | E11.40 | Type 2 diabetes mellitus with diabetic neuropathy, unspecified |
|  | I70-I79 | Diseases of arteries, arterioles and capillaries |
| Ophthalmology utilisation |  |  |
|  | 92002 | Ophthalmological services: medical examination and evaluation with initiation of diagnostic and treatment program; intermediate, new patient |
|  | 92004 | Ophthalmological services: medical examination and evaluation with initiation of diagnostic and treatment program; comprehensive, new patient, 1 or more visits |
|  | 92012 | Ophthalmological services: medical examination and evaluation, with initiation or continuation of diagnostic and treatment program; intermediate, established patient |
|  | 92014 | Ophthalmological services: medical examination and evaluation, with initiation or continuation of diagnostic and treatment program; comprehensive, established patient, 1 or more visits |
| Medications |  |  |
|  | C10 | Lipid modifying agents |
|  | A10A | Insulins and analogues |
|  | A10B | Blood glucose lowering drugs, excl insulins |
| Laboratory values |  |  |
|  | 9037 (TriNetX curated) | Hemoglobin A1c/Hemoglobin total in Blood |
|  | 9083 (TriNetX curated) | Body mass index (BMI) |
|  | 9000 (TriNetX curated) | Cholesterol [Mass/volume] in Serum or Plasma |
| Outcomes |  |  |
|  | E11.31, E11.32, E11.33, E11.34, E11.35 | Type 2 diabetes mellitus with any diabetic retinopathy, including unspecified |
|  | E11.32, E11.33, E11.34 | Type 2 diabetes mellitus with non-proliferative diabetic retinopathy |
|  | E11.35 | Type 2 diabetes mellitus with proliferative diabetic retinopathy |
|  | H33, H33.20, H33.23 | Retinal detachment |
|  | H43.13, H43.399 | Vitreous hemorrhage |
|  | H54, H44.52 | Blindness or low vision |
|  | E11.311, E11.321, E11.331, E11.341 | Type 2 diabetes mellitus with macular oedema |
|  | 67028 | Intravitreal injection |
|  | 67228 | Panretinal photocoagulation |
|  | 67113, 67108, 67036, 67040, 67041, 67042, 67043, 67039 | Pars plana vitrectomy |
|  | 92134 | Scanning computerized ophthalmic diagnostic imaging, posterior segment, with interpretation and report (OCT) |
|  | 92250 | Fundus photography |
|  | 92235 | Fluorescein angiography |
| Nonadherence |  |  |
|  | Z91.12 | Patient underdosing or noncompliance of medication regimen |
|  | Z91.13 | Patient’s unintentional underdosing of medication (e.g., forgetting or misunderstanding instructions) |
|  | Z91.14 | Other noncompliance with medication regimen (e.g., irregular dosing not otherwise specified) |
|  | Z91.19 | Noncompliance with other medical treatment/regimen, such as refusal of non-pharmacologic therapy (e.g., refusal or failure to comply with non-pharmacologic medical treatments like follow-up visits, diet, glucose monitoring) |
|  | Z53.20 | Procedure or treatment not carried out due to patient decision for unspecified reasons (e.g., refusal without reason given) |
|  | Z53.21 | Procedure or treatment not carried out because patient left before being seen (e.g., left without being seen in clinic/ED) |

| **Supplementary Table 2. Social Deprivation and Cumulative Incidence Rates of Diabetic Retinopathy Outcomes in 62,786 Patients with Type 2 Diabetes** | | | | | | |
| --- | --- | --- | --- | --- | --- | --- |
|  | **1 year** | | **5 years** | | **10 years** | |
| **Outcome** | **Socially Non-Deprived, n (%)** | **Socially Deprived, n (%)** | **Socially Non-Deprived, n (%)** | **Socially Deprived, n (%)** | **Socially Non-Deprived, n (%)** | **Socially Deprived, n (%)** |
| *DR incidence* |  |  |  |  |  |  |
| Any DR | 1,339 (2.1%) | 1,736 (2.8%) | 2,757 (4.4%) | 3,833 (6.1%) | 3,064 (4.9%) | 4,735 (7.5%) |
| NPDR | 359 (0.6%) | 567 (0.9%) | 1,010 (1.6%) | 1,652 (2.6%) | 1,303 (2.1%) | 2,346 (3.7%) |
| PDR | 171 (0.3%) | 237 (0.4%) | 371 (0.6%) | 658 (1.1%) | 484 (0.8%) | 882 (1.4%) |
| *Sight-threatening complications* |  |  |  |  |  |  |
| RD | 116 (0.2%) | 163 (0.3%) | 290 (0.5%) | 429 (0.7%) | 357 (0.6%) | 568 (0.9%) |
| VH | 103 (0.2%) | 133 (0.2%) | 221 (0.4%) | 421 (0.7%) | 290 (0.5%) | 533 (0.9%) |
| Blindness or low vision | 326 (0.5%) | 656 (1.1%) | 914 (1.5%) | 1,947 (3.1%) | 1,084 (1.7%) | 2,487 (4.0%) |
| Macular oedema | 302 (0.5%) | 372 (0.6%) | 700 (1.1%) | 1,060 (1.7%) | 858 (1.4%) | 1,375 (2.2%) |
| *DR treatment* |  |  |  |  |  |  |
| IVI | 225 (0.4%) | 299 (0.5%) | 375 (0.6%) | 536 (0.9%) | 468 (0.8%) | 783 (1.3%) |
| PRP | 161 (0.3%) | 170 (0.3%) | 243 (0.4%) | 357 (0.6%) | 306 (0.5%) | 506 (0.8%) |
| PPV | 123 (0.2%) | 147 (0.2%) | 227 (0.4%) | 343 (0.6%) | 273 (0.4%) | 470 (0.8%) |
| *Diagnostic imaging* |  |  |  |  |  |  |
| OCT | 860 (1.4%) | 1,107 (1.8%) | 1,709 (2.7%) | 2,433 (3.9%) | 2,237 (3.6%) | 3,523 (5.6%) |
| Fundus photography | 407 (0.7%) | 482 (0.8%) | 956 (1.5%) | 1,265 (2.0%) | 1,325 (2.1%) | 1,822 (2.9%) |
| Fluorescein angiography | 106 (0.2%) | 121 (0.2%) | 220 (0.4%) | 304 (0.5%) | 279 (0.4%) | 420 (0.7%) |
| Nonadherence to treatment |  |  |  |  |  |  |
| Nonadherence (Overall) | 2,077 (3.3%) | 8,082 (12.9%) | 5,467 (8.7%) | 17,368 (27.7%) | 5,630 (9.0%) | 19,579 (31.2%) |
| Nonadherence to medications | 1,131 (1.8%) | 4,499 (7.2%) | 2,970 (4.7%) | 10,461 (16.7%) | 2,937 (4.7%) | 11,769 (18.7%) |
| Nonpersistence to procedures | 569 (0.9%) | 2,101 (3.4%) | 1,799 (2.9%) | 5,414 (8.6%) | 1,902 (3.0%) | 6,498 (10.4%) |
| Abbreviations: DR, diabetic retinopathy; IVI, intravitreal injection; NPDR, non-proliferative diabetic retinopathy; OCT, optical coherence tomography; PDR, proliferative diabetic retinopathy; PPV, pars plana vitrectomy; PRP, panretinal photocoagulation; RD, retinal detachment; VH, vitreous hemorrhage. | | | | | | |

| **Supplementary Table 3. Social Deprivation and Cumulative Incidence Rates of Diabetic Retinopathy Outcomes in 35,584 Patients with Type 2 Diabetes and Documented Adherence** | | | | | | |
| --- | --- | --- | --- | --- | --- | --- |
|  | **1 year** | | **5 years** | | **10 years** | |
| **Outcome** | **Socially Non-Deprived, n (%)** | **Socially Deprived, n (%)** | **Socially Non-Deprived, n (%)** | **Socially Deprived, n (%)** | **Socially Non-Deprived, n (%)** | **Socially Deprived, n (%)** |
| *DR incidence* |  |  |  |  |  |  |
| Any DR | 720 (2.0%) | 929 (2.6%) | 1,350 (3.8%) | 1,922 (5.4%) | 1,545 (4.3%) | 2,416 (6.8%) |
| NPDR | 184 (0.5%) | 303 (0.9%) | 512 (1.4%) | 860 (2.4%) | 636 (1.8%) | 1,240 (3.5%) |
| PDR | 82 (0.2%) | 124 (0.3%) | 170 (0.5%) | 306 (0.9%) | 216 (0.6%) | 414 (1.2%) |
| *Sight-threatening complications* |  |  |  |  |  |  |
| RD | 57 (0.2%) | 90 (0.3%) | 142 (0.4%) | 221 (0.6%) | 198 (0.6%) | 292 (0.8%) |
| VH | 49 (0.1%) | 77 (0.2%) | 112 (0.3%) | 224 (0.6%) | 134 (0.4%) | 268 (0.8%) |
| Blindness or low vision | 183 (0.5%) | 324 (0.9%) | 469 (1.3%) | 893 (2.5%) | 532 (1.5%) | 1,147 (3.2%) |
| Macular oedema | 164 (0.5%) | 194 (0.5%) | 356 (1.0%) | 560 (1.6%) | 450 (1.3%) | 714 (2.0%) |
| *DR treatment* |  |  |  |  |  |  |
| IVI | 174 (0.5%) | 183 (0.5%) | 212 (0.6%) | 318 (0.9%) | 299 (0.8%) | 437 (1.2%) |
| PRP | 69 (0.2%) | 87 (0.2%) | 101 (0.3%) | 160 (0.4%) | 124 (0.3%) | 214 (0.6%) |
| PPV | 85 (0.2%) | 82 (0.2%) | 131 (0.4%) | 169 (0.5%) | 165 (0.5%) | 228 (0.6%) |
| *Diagnostic imaging* |  |  |  |  |  |  |
| OCT | 517 (1.5%) | 657 (1.8%) | 900 (2.5%) | 1,376 (3.9%) | 1,247 (3.5%) | 1,975 (5.6%) |
| Fundus photography | 253 (0.7%) | 286 (0.8%) | 508 (1.4%) | 731 (2.1%) | 750 (2.1%) | 1,021 (2.9%) |
| Fluorescein angiography | 64 (0.2%) | 64 (0.2%) | 121 (0.3%) | 174 (0.5%) | 154 (0.4%) | 227 (0.6%) |
| Abbreviations: DR, diabetic retinopathy; IVI, intravitreal injection; NPDR, non-proliferative diabetic retinopathy; OCT, optical coherence tomography; PDR, proliferative diabetic retinopathy; PPV, pars plana vitrectomy; PRP, panretinal photocoagulation; RD, retinal detachment; VH, vitreous hemorrhage. | | | | | | |

| **Supplementary Table 4. Effects of Social Deprivation and Sex on Diabetic Retinopathy Outcomes in Patients with Type 2 Diabetes over 10-Year Follow-Up** | | |
| --- | --- | --- |
|  | **Female** | **Male** |
| **Outcome** | **HR (95% CI)** | **HR (95% CI)** |
| *Diabetic retinopathy incidence* | | |
| Any DR | 1.53 (1.43, 1.64) | 1.38 (1.30, 1.48) |
| NPDR | 1.79 (1.61, 1.98) | 1.59 (1.44, 1.75) |
| PDR | 1.77 (1.49, 2.11) | 1.69 (1.45, 1.97) |
| *Sight-threatening complications* | | |
| RD | 1.57 (1.28, 1.93) | 1.32 (1.10, 1.59) |
| VH | 1.71 (1.40, 2.10) | 1.68 (1.34, 2.10) |
| Blindness or low vision | 2.18 (1.95, 2.43) | 2.10 (1.90, 2.33) |
| Macular oedema | 1.51 (1.33, 1.72) | 1.34 (1.19, 1.52) |
| *DR treatment* | | |
| IVI | 1.54 (1.29, 1.83) | 1.37 (1.18, 1.59) |
| PRP | 1.70 (1.35, 2.13) | 1.39 (1.16, 1.68) |
| PPV | 1.63 (1.29, 2.07) | 1.52 (1.24, 1.85) |
| *Diagnostic imaging* | | |
| OCT | 1.45 (1.35, 1.56) | 1.35 (1.25, 1.46) |
| Fundus photography | 1.31 (1.19, 1.45) | 1.23 (1.10, 1.36) |
| Fluorescein angiography | 1.43 (1.14, 1.78) | 1.33 (1.08, 1.65) |
| Abbreviations: CI, confidence interval; DR, diabetic retinopathy; HR, hazard ratio; IVI, intravitreal injection; NPDR, non-proliferative diabetic retinopathy; OCT, optical coherence tomography; PDR, proliferative diabetic retinopathy; PPV, pars plana vitrectomy; PRP, panretinal photocoagulation; RD, retinal detachment; VH, vitreous hemorrhage. | | |

| **Supplementary Table 5. Effects of Social Deprivation and Race/Ethnicity on Diabetic Retinopathy Outcomes in Patients with Type 2 Diabetes over 10 Year Follow-Up** | | | | |
| --- | --- | --- | --- | --- |
|  | **Non-Hispanic White** | **Non-Hispanic Black or African American** | **Non-White Hispanic or Latino** | **Non-Hispanic Asian** |
| **Outcome** | **HR (95% CI)** | **HR (95% CI)** | **HR (95% CI)** | **HR (95% CI)** |
| *Diabetic retinopathy incidence* | | | | |
| Any DR | 1.52 (1.40, 1.65) | 1.26 (1.17, 1.37) | 1.63 (1.36, 1.95) | 1.33 (1.04, 1.70) |
| NPDR | 1.73 (1.53, 1.95) | 1.50 (1.34, 1.69) | 2.36 (1.77, 3.14) | 1.14 (0.79, 1.65) |
| PDR | 1.41 (1.14, 1.75) | 1.44 (1.18, 1.76) | 2.19 (1.50, 3.21) | 2.27 (1.05, 4.90) |
| *Sight-threatening complications* | | | | |
| RD | 1.21 (0.95, 1.53) | 1.53 (1.22, 1.93) | 1.69 (1.01, 2.84) | 1.09 (0.43, 2.77) |
| VH | 2.10 (1.60, 2.76) | 1.64 (1.26, 2.14) | 1.57 (0.87, 2.86) | 1.41 (0.55, 3.65) |
| Blindness or low vision | 2.05 (1.82, 2.31) | 1.86 (1.65, 2.10) | 2.25 (1.63, 3.10) | 2.44 (1.54, 3.87) |
| Macular oedema | 1.64 (1.40, 1.94) | 1.14 (0.99, 1.33) | 1.64 (1.17, 2.32) | 1.67 (0.92, 3.00) |
| *DR treatment* | | | | |
| IVI | 1.38 (1.13, 1.68) | 1.11 (0.91, 1.37) | 1.41 (0.98, 2.02) | 2.81 (1.12, 7.06) |
| PRP | 1.77 (1.32, 2.39) | 1.13 (0.89, 1.43) | 2.00 (1.26, 3.17) | 1.27 (0.36, 4.52) |
| PPV | 1.52 (1.15, 2.02) | 1.24 (0.95, 1.63) | 1.61 (1.02, 2.55) | 1.17 (0.51, 2.68) |
| *Diagnostic imaging* | | | | |
| OCT | 1.50 (1.37, 1.64) | 1.41 (1.29, 1.55) | 1.27 (1.05, 1.53) | 1.21 (0.86, 1.71) |
| Fundus photography | 1.23 (1.10, 1.39) | 1.34 (1.18, 1.51) | 1.26 (0.99, 1.60) | 1.10 (0.66, 1.83) |
| Fluorescein angiography | 1.37 (1.04, 1.81) | 1.48 (1.13, 1.94) | 1.68 (1.03, 2.76) | 1.03 (0.31, 3.38) |
| Abbreviations: CI, confidence interval; DR, diabetic retinopathy; HR, hazard ratio; IVI, intravitreal injection; NPDR, non-proliferative diabetic retinopathy; OCT, optical coherence tomography; PDR, proliferative diabetic retinopathy; PPV, pars plana vitrectomy; PRP, panretinal photocoagulation; RD, retinal detachment; VH, vitreous hemorrhage. | | | | |

| **Supplementary Table 6. Effects of Social Deprivation and Age on Diabetic Retinopathy Outcomes in Patients with Type 2 Diabetes over 10-Year Follow-Up** | | | |
| --- | --- | --- | --- |
|  | **18-39 years** | **40-64 years** | **65+ years** |
| **Outcome** | **HR (95% CI)** | **HR (95% CI)** | **HR (95% CI)** |
| *Diabetic retinopathy incidence* | | | |
| Any DR | 1.50 (1.14, 1.98) | 1.35 (1.29, 1.42) | 1.32 (1.23, 1.42) |
| NPDR | 1.48 (1.19, 1.84) | 1.57 (1.46, 1.69) | 1.54 (1.37, 1.74) |
| PDR | 1.59 (1.13, 2.22) | 1.52 (1.35, 1.72) | 1.59 (1.27, 1.99) |
| *Sight-threatening complications* | | | |
| RD | 1.21 (0.84, 1.74) | 1.49 (1.28, 1.73) | 1.39 (1.08, 1.78) |
| VH | 1.28 (0.82, 2.01) | 1.74 (1.48, 2.05) | 1.87 (1.39, 2.53) |
| Blindness or low vision | 1.80 (1.44, 2.25) | 2.05 (1.89, 2.23) | 1.94 (1.72, 2.20) |
| Macular oedema | 1.21 (0.90, 1.63) | 1.43 (1.30, 1.58) | 1.46 (1.25, 1.71) |
| *DR treatment* | | | |
| IVI | 1.61 (1.06, 2.44) | 1.51 (1.33, 1.71) | 1.32 (1.10, 1.58) |
| PRP | 1.42 (0.92, 2.19) | 1.73 (1.48, 2.02) | 1.24 (0.93, 1.67) |
| PPV | 1.62 (0.99, 2.63) | 1.35 (1.16, 1.59) | 1.50 (1.12, 2.00) |
| *Diagnostic imaging* | | | |
| OCT | 1.32 (1.10, 1.57) | 1.39 (1.31, 1.48) | 1.26 (1.16, 1.36) |
| Fundus photography | 1.19 (0.98, 1.45) | 1.24 (1.14, 1.34) | 1.17 (1.02, 1.33) |
| Fluorescein angiography | 1.51 (0.85, 2.71) | 1.47 (1.23, 1.75) | 1.35 (1.05, 1.75) |
| Abbreviations: CI, confidence interval; DR, diabetic retinopathy; HR, hazard ratio; IVI, intravitreal injection; NPDR, non-proliferative diabetic retinopathy; OCT, optical coherence tomography; PDR, proliferative diabetic retinopathy; PPV, pars plana vitrectomy; PRP, panretinal photocoagulation; RD, retinal detachment; VH, vitreous hemorrhage. | | | |

| **Supplemental Table 7. Effect of Social Deprivation on Diabetic Retinopathy Outcomes in Patients with Type 2 Diabetes Mellitus over 1-, 5-, and 10-year Follow-up** | | | |
| --- | --- | --- | --- |
|  | **Housing Instability vs. Financial Hardship** | **Low Health Literacy vs. Financial Hardship** | **Low Health Literacy vs. Housing Instability** |
| **Outcome** | **HR (95% CI)** | **HR (95% CI)** | **HR (95% CI)** |
| *DR incidence* | | | |
| Any DR | 0.76 (0.71, 0.80) | 0.85 (0.77, 0.93) | 1.16 (1.04, 1.30) |
| NPDR | 0.74 (0.68, 0.79) | 0.92 (0.81, 1.03) | 1.18 (1.03, 1.36) |
| PDR | 0.65 (0.57, 0.74) | 0.78 (0.65, 0.93) | 1.37 (1.09, 1.72) |
| *Sight-threatening complications* |  |  |  |
| RD | 0.74 (0.63, 0.86) | 0.75 (0.58, 0.97) | 1.19 (0.87, 1.64) |
| VH | 0.72 (0.61, 0.84) | 0.58 (0.44, 0.77) | 0.89 (0.64, 1.25) |
| Blindness or low vision | 0.89 (0.83, 0.96) | 0.83 (0.74, 0.95) | 0.95 (0.82, 1.09) |
| Macular oedema | 0.71 (0.65, 0.79) | 0.74 (0.62, 0.88) | 1.01 (0.82, 1.24) |
| *DR treatment* |  |  |  |
| IVI | 0.64 (0.55, 0.74) | 0.76 (0.63, 0.91) | 1.29 (1.01, 1.65) |
| PRP | 0.62 (0.51, 0.76) | 0.82 (0.64, 1.04) | 1.84 (1.30, 2.59) |
| PPV | 0.79 (0.66, 0.94) | 0.78 (0.60, 1.02) | 1.33 (0.96, 1.85) |
| *Diagnostic imaging* |  |  |  |
| OCT | 0.74 (0.69, 0.78) | 0.73 (0.65, 0.81) | 1.00 (0.88, 1.13) |
| Fundus photography | 0.79 (0.72, 0.86) | 0.46 (0.38, 0.55) | 0.64 (0.52, 0.79) |
| Fluorescein angiography | 0.66 (0.55, 0.81) | 0.60 (0.43, 0.83) | 0.88 (0.58, 1.34) |
| *Nonadherence to treatment* |  |  |  |
| Nonadherence (Overall) | 1.45 (1.41, 1.48) | 0.70 (0.67, 0.74) | 0.52 (0.49, 0.55) |
| Nonadherence to medications | 1.31 (1.27, 1.35) | 0.69 (0.64, 0.74) | 0.57 (0.53, 0.62) |
| Nonadherence to procedures | 1.61 (1.54, 1.68) | 0.74 (0.67, 0.83) | 0.47 (0.42, 0.52) |
| Each comparison is relative to the first group listed (e.g., housing instability vs. financial hardship uses housing instability as the reference). HRs < 1 indicate lower risk or rates in the reference group. | | | |
| Abbreviations: CI, confidence interval; DR, diabetic retinopathy; HR, hazard ratio; IVI, intravitreal injection; NPDR, non-proliferative diabetic retinopathy; OCT, optical coherence tomography; PDR, proliferative diabetic retinopathy; PPV, pars plana vitrectomy; PRP, panretinal photocoagulation; RD, retinal detachment; VH, vitreous hemorrhage. | | | |

| **Supplementary Table 8. Baseline Characteristics of Patients with Type 2 Diabetes Mellitus at First Ophthalmology Encounter Stratified by Social Deprivation Status Before and After Propensity Score Matching** | | | | | | | |
| --- | --- | --- | --- | --- | --- | --- | --- |
|  | **Before propensity score matching, No. (%)** | | |  | **After propensity score matching, No. (%)** | | |
| **Characteristic** | **Socially deprived (N = 15,273)** | **Socially Non-Deprived (N = 650,910)** | **SMD** |  | **Socially deprived (N = 15,271)** | **Socially Non-Deprived (N = 15,271)** | **SMD** |
| **Demographics** |  |  |  |  |  |  |  |
| Age at index, years, mean ± SD | 55.3 ± 12.3 | 60.4 ± 12.7 | 0.409 |  | 55.3 ± 12.3 | 55.1 ± 13.0 | 0.014 |
| Female | 8,397 (55.0) | 347,568 (53.4) | 0.032 |  | 8,396 (55.0) | 8,568 (56.1) | 0.023 |
| White | 6,612 (43.3) | 346,917 (53.3) | 0.201 |  | 6,612 (43.3) | 6,566 (43.0) | 0.006 |
| American Indian or Alaska Native | 122 (0.8) | 3,033 (0.5) | 0.042 |  | 122 (0.8) | 102 (0.7) | 0.015 |
| Native Hawaiian or Other Pacific Islander | 43 (0.3) | 1,972 (0.3) | 0.004 |  | 43 (0.3) | 31 (0.2) | 0.016 |
| Hispanic or Latino | 2,178 (14.3) | 88,594 (13.6) | 0.019 |  | 2,176 (14.2) | 2,211 (14.5) | 0.007 |
| Black or African American | 6,315 (41.3) | 140,804 (21.6) | 0.434 |  | 6,313 (41.3) | 6,495 (42.5) | 0.024 |
| Asian | 312 (2.0) | 28,494 (4.4) | 0.133 |  | 312 (2.0) | 284 (1.9) | 0.013 |
| **Comorbidities** |  |  |  |  |  |  |  |
| Chronic kidney disease | 2,514 (16.5) | 75,318 (11.6) | 0.141 |  | 2,514 (16.5) | 2,369 (15.5) | 0.026 |
| Hyperlipidaemia | 8,545 (55.9) | 287,662 (44.2) | 0.237 |  | 8,544 (55.9) | 8,668 (56.8) | 0.016 |
| Ischaemic heart diseases | 3,754 (24.6) | 115,928 (17.8) | 0.166 |  | 3,753 (24.6) | 3,646 (23.9) | 0.016 |
| Hypertensive diseases | 11,598 (75.9) | 406,628 (62.5) | 0.295 |  | 11,597 (75.9) | 11,580 (75.8) | 0.003 |
| Chronic lower respiratory diseases | 6,063 (39.7) | 141,238 (21.7) | 0.398 |  | 6,061 (39.7) | 5,861 (38.4) | 0.027 |
| Tobacco use | 2,411 (15.8) | 21,441 (3.3) | 0.435 |  | 2,409 (15.8) | 2,282 (14.9) | 0.023 |
| Cerebrovascular diseases | 2,388 (15.6) | 66,374 (10.2) | 0.163 |  | 2,388 (15.6) | 2,200 (14.4) | 0.034 |
| Type 2 diabetes mellitus with diabetic neuropathy | 1,173 (7.7) | 30,534 (4.7) | 0.124 |  | 1,172 (7.7) | 1,089 (7.1) | 0.021 |
| Diseases of arteries, arterioles and capillaries | 2,824 (18.5) | 82,126 (12.6) | 0.163 |  | 2,823 (18.5) | 2,594 (17.0) | 0.039 |
| **Ophthalmology care utilisation** |  |  |  |  |  |  |  |
| General ophthalmological services | 5,264 (34.5) | 210,992 (32.4) | 0.043 |  | 5,263 (34.5) | 5,083 (33.3) | 0.025 |
| **Medications** |  |  |  |  |  |  |  |
| Lipid modifying agents | 8,732 (57.2) | 330,707 (50.8) | 0.128 |  | 8,731 (57.2) | 8,742 (57.2) | 0.001 |
| Insulins and analogues | 5,429 (35.5) | 149,056 (22.9) | 0.281 |  | 5,428 (35.5) | 5,355 (35.1) | 0.01 |
| Blood glucose lowering agents | 7,829 (51.3) | 307,532 (47.2) | 0.08 |  | 7,829 (51.3) | 8,010 (52.5) | 0.024 |
| **Laboratory values, mean ± SD** |  |  |  |  |  |  |  |
| Hemoglobin A1c (%) | 7.2 ± 2.1 | 7.1 ± 1.9 | 0.057 |  | 7.2 ± 2.1 | 7.1 ± 2.0 | 0.056 |
| BMI (kg/m^2^) | 34.9 ± 9.3 | 33.1 ± 8.0 | 0.206 |  | 34.9 ± 9.3 | 34.3 ± 8.5 | 0.072 |
| Total cholesterol (mg/dL) | 176.2 ± 51.1 | 173.0 ± 48.7 | 0.064 |  | 176.2 ± 51.1 | 172.8 ± 51.4 | 0.065 |
| Abbreviations: SMD, standardized mean difference; SD, standard deviation; BMI, body mass index. | | | | | | | |

| **Supplementary Table 9. Effect of Social Deprivation on Diabetic Retinopathy Outcomes in Patients with Type 2 Diabetes Mellitus Without Documented Nonadherence at First Ophthalmology Encounter over 1-, 5-, and 10-year follow-up** | | | |
| --- | --- | --- | --- |
|  | **1 year** | **5 years** | **10 years** |
| **Outcome** | **HR (95% CI)** | **HR (95% CI)** | **HR (95% CI)** |
| *Diabetic retinopathy incidence* | | | |
| Any DR | 1.05 (0.97, 1.13) | 1.04 (0.98, 1.11) | 1.13 (1.07, 1.20) |
| NPDR | 1.19 (0.97, 1.45) | 1.15 (1.03, 1.28) | 1.30 (1.18, 1.43) |
| PDR | 1.05 (0.81, 1.36) | 1.18 (0.99, 1.40) | 1.32 (1.12, 1.55) |
| *Sight-threatening complications* | | | |
| RD | 1.18 (0.90, 1.56) | 1.15 (0.96, 1.38) | 1.09 (0.93, 1.29) |
| VH | 1.40 (1.02, 1.91) | 1.28 (1.06, 1.55) | 1.32 (1.11, 1.58) |
| Blindness or low vision | 1.66 (1.36, 2.03) | 1.78 (1.58, 2.01) | 1.74 (1.57, 1.94) |
| Macular oedema | 0.93 (0.76, 1.15) | 0.95 (0.84, 1.08) | 1.08 (0.96, 1.22) |
| *DR treatment* | | | |
| IVI | 1.08 (0.94, 1.25) | 1.04 (0.92, 1.16) | 1.15 (1.03, 1.28) |
| PRP | 1.13 (0.93, 1.36) | 1.31 (1.12, 1.54) | 1.31 (1.13, 1.51) |
| PPV | 1.01 (0.82, 1.24) | 1.18 (1.00, 1.40) | 1.19 (1.02, 1.39) |
| *Diagnostic imaging* | | | |
| OCT | 0.99 (0.92, 1.08) | 1.00 (0.94, 1.06) | 1.05 (0.99, 1.11) |
| Fundus photography | 1.02 (0.90, 1.17) | 0.99 (0.91, 1.08) | 1.00 (0.93, 1.09) |
| Fluorescein angiography | 1.02 (0.83, 1.25) | 0.87 (0.75, 1.02) | 1.01 (0.87, 1.17) |
| Abbreviations: CI, confidence interval; DR, diabetic retinopathy; HR, hazard ratio; IVI, intravitreal injection; NPDR, non-proliferative diabetic retinopathy; OCT, optical coherence tomography; PDR, proliferative diabetic retinopathy; PPV, pars plana vitrectomy; PRP, panretinal photocoagulation; RD, retinal detachment; VH, vitreous hemorrhage. | | | |

**Supplementary Figure 1. Diabetic Retinopathy–Free Survival at 3 Years in Patients with Type 2 Diabetes**

**
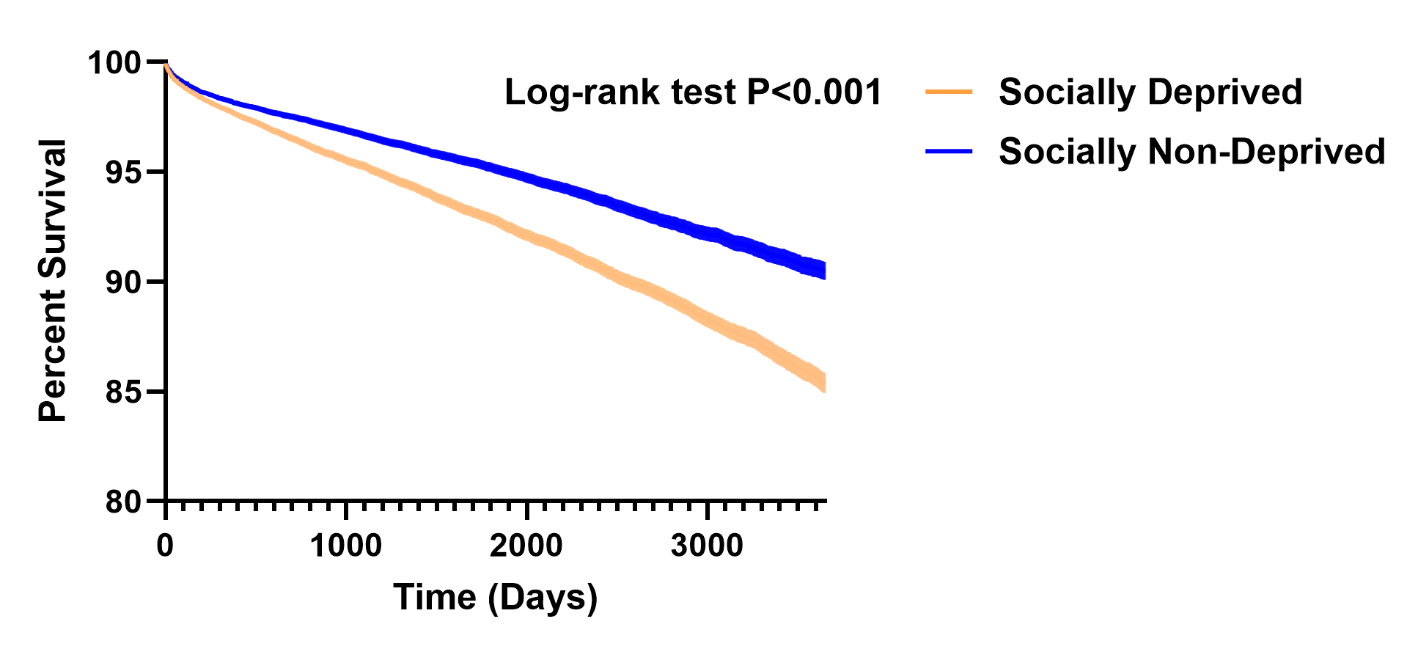
**

Kaplan-Meier survival analysis of diabetic retinopathy–free survival over 10 years in patients with type 2 diabetes, stratified by social deprivation status. The y-axis indicates the proportion of participants remaining free from any stage of DR, while the x-axis represents time in days from the index event. Patients were right-censored at the earliest of the following: (1) diagnosis of DR, (2) death, (3) loss to follow-up after the index event, or (4) end of the 10-year observation period.

**Supplementary Figure 2. Diabetic Retinopathy–Free Survival at 3 Years in Patients with Type 2 Diabetes and Documented Adherence**

**
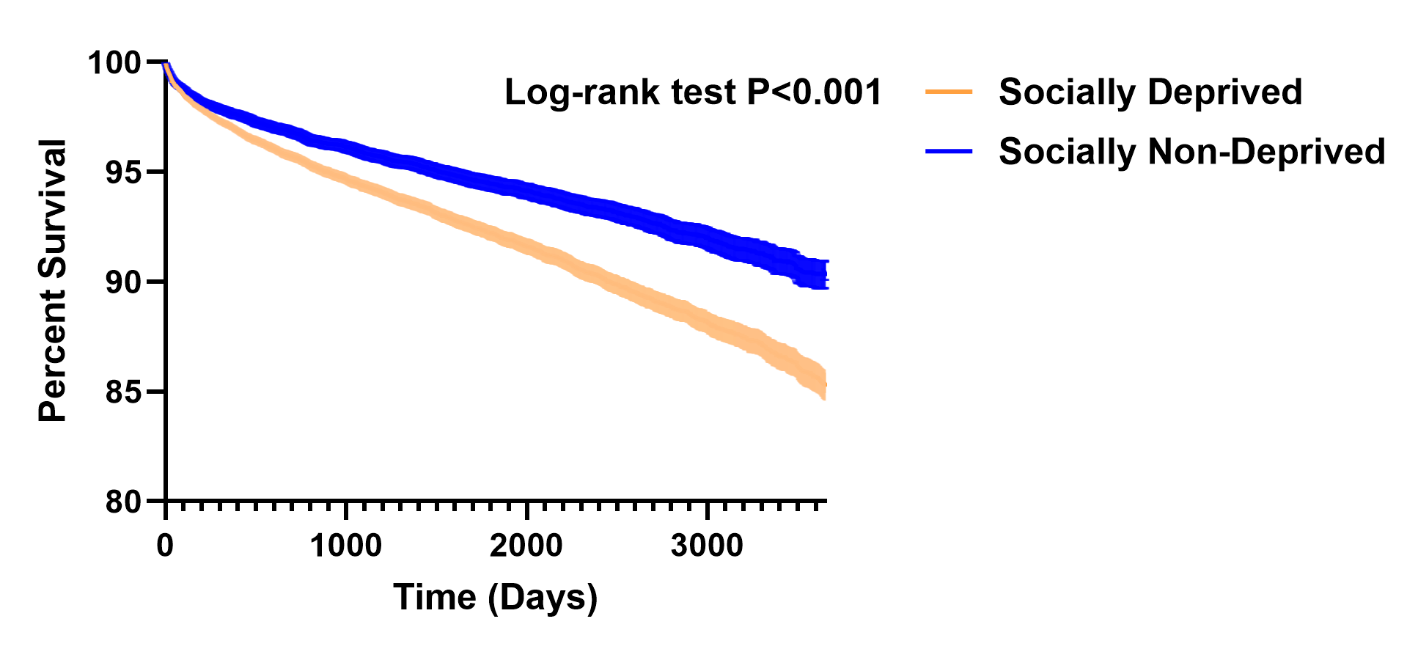
**

Kaplan-Meier survival analysis of diabetic retinopathy–free survival over 10 years in patients with type 2 diabetes with documented adherence (defined by lack of documented nonadherence diagnoses), stratified by social deprivation status. The y-axis indicates the proportion of participants remaining free from any stage of DR, while the x-axis represents time in days from the index event. Patients were right-censored at the earliest of the following: (1) diagnosis of DR, (2) death, (3) loss to follow-up after the index event, or (4) end of the 10-year observation period.
